# (3,4-Dimeth­oxy­phen­yl)[2-(thio­phen-2-ylcarbon­yl)phen­yl]methanone

**DOI:** 10.1107/S1600536812037336

**Published:** 2012-09-05

**Authors:** G. Ganesh, R. Sivasakthikumaran, E. Govindan, A. K. Mohana Krishnan, A. SubbiahPandi

**Affiliations:** a Department of Physics, S.M.K. Fomra Institute of Technology, Thaiyur, Chennai 603 103, India; b Department of Organic Chemistry, University of Madras, Guindy Campus, Chennai 600 025, India; c Department of Physics, Presidency College (Autonomous), Chennai 600 005, India

## Abstract

In the title compound, C_20_H_16_O_4_S, the thiophene ring makes dihedral angles of 72.9 (2) and 60.5 (2)°, respectively, with the dimethoxy benzene and phenyl rings. In the crystal, C—H⋯O hydrogen bonds link the mol­ecules into a *C*(9) chain along the *b* axis. The S and C atoms of the thio­phene ring are disordered over two sets of sites [site occupancies = 0.675 (3) and 0.325 (3)]. A short inter­molecular S⋯O contact [3.084 (2) Å] is observed in the crystal structure, which also features C—H⋯π inter­actions.

## Related literature

For background to thio­phene derivatives and their biological activity, see: Bonini *et al.* (2005 ▶); Khan *et al.* (2009 ▶); Brault *et al.* (2005 ▶); Isloora *et al.* (2010 ▶); Xia *et al.* (2010 ▶). For related structures, see: Asiri *et al.* (2010 ▶); Aslam *et al.* (2011 ▶).
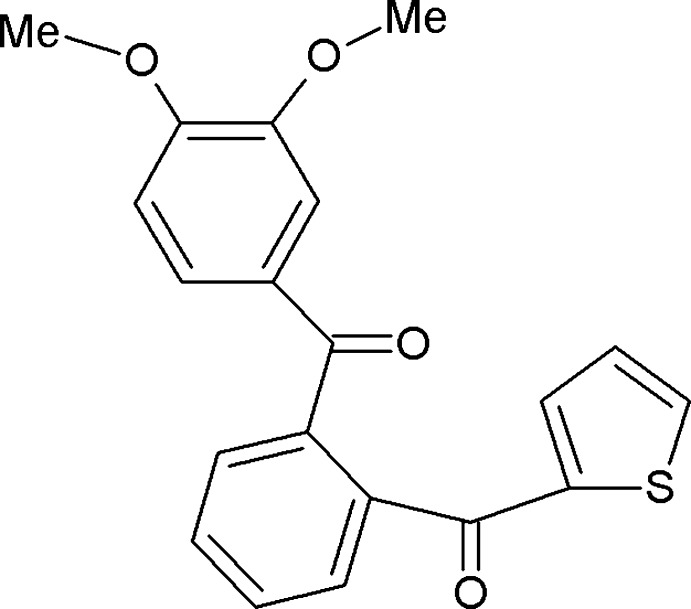


## Experimental

### 

#### Crystal data

C_20_H_16_O_4_S
*M* *_r_* = 352.40
Orthorhombic, 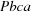

*a* = 15.7324 (6) Å
*b* = 10.7988 (5) Å
*c* = 20.4877 (11) Å
*V* = 3480.7 (3) Å^3^
*Z* = 8
Mo *K*α radiation
μ = 0.21 mm^−1^
*T* = 293 K
0.25 × 0.22 × 0.19 mm

#### Data collection

Bruker APEXII CCD area-detector diffractometer
Absorption correction: multi-scan (*SADABS*; Sheldrick, 1996 ▶) *T*_min_ = 0.950, *T*_max_ = 0.961
22015 measured reflections
4469 independent reflections
2968 reflections with *I* > 2σ(*I*)
*R*_int_ = 0.029

#### Refinement

*R*[*F* ^2^ > 2σ(*F* ^2^)] = 0.040
*wR*(*F* ^2^) = 0.107
*S* = 1.03
4469 reflections
236 parameters
4 restraints
H-atom parameters constrained
Δρ_max_ = 0.19 e Å^−3^
Δρ_min_ = −0.20 e Å^−3^

### 

Data collection: *APEX2* (Bruker, 2008 ▶); cell refinement: *SAINT* (Bruker, 2008 ▶); data reduction: *SAINT*; program(s) used to solve structure: *SHELXS97* (Sheldrick, 2008 ▶); program(s) used to refine structure: *SHELXL97* (Sheldrick, 2008 ▶); molecular graphics: *ORTEP-3* (Farrugia, 1997 ▶); software used to prepare material for publication: *SHELXL97* and *PLATON* (Spek, 2009 ▶).

## Supplementary Material

Crystal structure: contains datablock(s) global, I. DOI: 10.1107/S1600536812037336/bt5963sup1.cif

Structure factors: contains datablock(s) I. DOI: 10.1107/S1600536812037336/bt5963Isup2.hkl

Supplementary material file. DOI: 10.1107/S1600536812037336/bt5963Isup3.mol

Supplementary material file. DOI: 10.1107/S1600536812037336/bt5963Isup4.cml

Additional supplementary materials:  crystallographic information; 3D view; checkCIF report

## Figures and Tables

**Table 1:** Hydrogen-bond geometry (Å, °) *Cg*2 is the centroid of the C17/C19/C20/S1′/C18′ ring.

*D*—H⋯*A*	*D*—H	H⋯*A*	*D*⋯*A*	*D*—H⋯*A*
C3—H3⋯O4^i^	0.93	2.58	3.496 (2)	169
C1—H1*A*⋯*Cg*2^ii^	0.96	2.99	3.799 (3)	143

Symmetry codes: (i) 
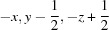
; (ii) 
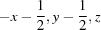
.

**Fig. 1.**
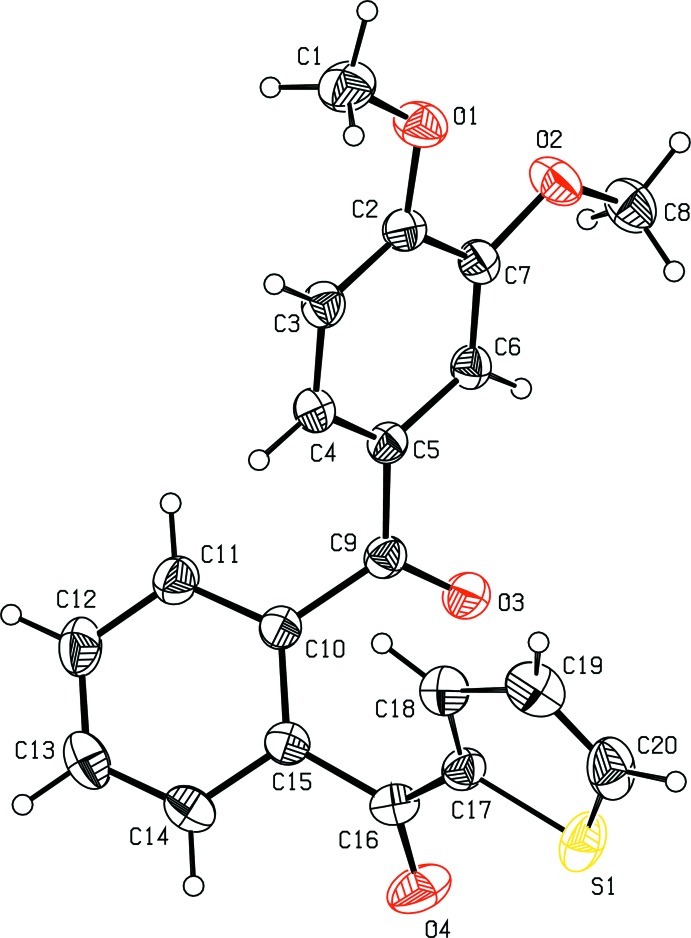
The molecular structure of the title compound, showing displacement ellipsoids drawn at the 30% probability level. H atoms are presented as a small spheres of arbitrary radius.

**Fig. 2.**
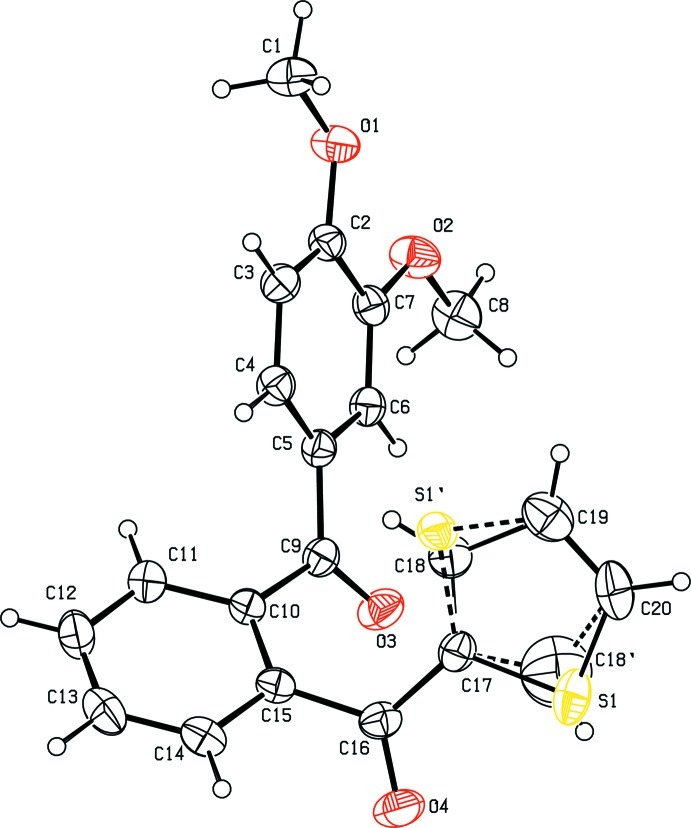
The molecular structure showing the major and minor occupied site of the disordered atoms.

## supplementary crystallographic information

### Comment

Thiophene derivatives exhibit anti-HIVPR inhibition (Bonini *et al.*, 2005) and antibreast cancer (Brault *et al.*, 2005) activity. In addition, some of the benzo[*b*]thiophene derivatives show significant antimicrobial and anti-inflammatory activity (Isloora *et al.*, 2010). Thiophene derivates have been viewed as significant compounds for applications in many fields (Xia *et al.*, 2010). Schiff bases are well known ligands in coordination chemistry with a wide range of biological activities (Khan *et al.*, 2009). Against this background, and in order to obtain detailed information on molecular the solid state, an X-ray study of the title compound was carried out.

X-Ray analysis confirms the molecular structure and atom connectivity as illustrated in Fig. 1. The bond lengths S1—C20 and O2—C8 are normal and comparable to the corresponding values observed in the related structure of (2E)-1-(2,5-Dimethyl-3-thienyl)-3-(2-methoxyphenyl)prop-2-en-1-one (Asiri *et al.*, 2010). The thiophene ring system makes dihedral angles of 72.9 (2) ° and 60.5 (2) °, respectively, with the dimethoxy benzene and the phenyl ring. The atoms O1 and O2 are deviated by -0.002 (1) Å and -0.010 (1) Å from the least squares plane of the C2—C7 ring. The atoms C18 and S1 of the thiophene ring are disordered over two positions [site occupancies = 0.648 (2) and 0.352 (2)].

The atom C3 acts as a donor to the atom O4 of the neighbour molecule at (-*x*,-1/2 + *y*, -*z*). This hydrogen bond is involved in a motif C(9) chain along *b* axis. Interestingly, a short non-hydrogen intermolecular contact between S1···O2 [3.084 (2) Å] at (1/2 - *x*,1 - *y*,-1/2 + *z*) was observed in the crystal structure. In addition to van der Waals interactions, the crystal packing is stabilized by C—H···π interaction between one of the methyl H atom (H1A) and the centroid (*Cg*2) of the thiophene ring (Table 1).

### Experimental

To a stirred suspension of benzoic (1 g, 3.44 mmol) in dry THF (20 ml) lead tetra acetate (1.5 g, 3.42 mmol) was added and refluxed at 50 °C for half an hour. The reaction mixture was then poured into water (200 ml) and extracted with ethyl acetate (2x20 ml), washed with brine solution and dried (Na_2_SO_4_). The removal of solvent *in vacuo* afforded the crude product upon crystallization from methanol furnished the title compound as a color less solid.

### Refinement

The S and C atoms of the thiophene ring are disordered over two positions (C18/C18' and S1/S1') with refined occupancies of 0.675 (3) and 0.325 (3). Equivalent C-C and C-S distances involving the disordered atoms were restrained to be equal with an effective e.s.d. of 0.01Å. The disordered C atoms were only isotropically refined. All H atoms were fixed geometrically and allowed to ride on their parent C atoms, with C—H distances fixed in the range 0.93–0.97 Å with *U*_iso_(H) = 1.5*U*_eq_(C) for methyl H 1.2*U*_eq_(C) for other H atoms.

### Crystal data

**Table:**

C_20_H_16_O_4_S	*F*(000) = 1472
*M**_r_* = 352.40	*D*_x_ = 1.345 Mg m^−^^3^
Orthorhombic, *P**b**c**a*	Mo *K*α radiation, λ = 0.71073 Å
Hall symbol: -P 2ac 2ab	Cell parameters from 4469 reflections
*a* = 15.7324 (6) Å	θ = 2.4–28.6°
*b* = 10.7988 (5) Å	µ = 0.21 mm^−^^1^
*c* = 20.4877 (11) Å	*T* = 293 K
*V* = 3480.7 (3) Å^3^	Block, white crystalline
*Z* = 8	0.25 × 0.22 × 0.19 mm

### Data collection

**Table:**

Bruker APEXII CCD area-detector diffractometer	4469 independent reflections
Radiation source: fine-focus sealed tube	2968 reflections with *I* > 2σ(*I*)
Graphite monochromator	*R*_int_ = 0.029
ω and φ scans	θ_max_ = 28.6°, θ_min_ = 2.4°
Absorption correction: multi-scan (*SADABS*; Sheldrick, 1996)	*h* = −21→12
*T*_min_ = 0.950, *T*_max_ = 0.961	*k* = −14→13
22015 measured reflections	*l* = −26→27

### Refinement

**Table:**

Refinement on *F*^2^	Secondary atom site location: difference Fourier map
Least-squares matrix: full	Hydrogen site location: inferred from neighbouring sites
*R*[*F*^2^ > 2σ(*F*^2^)] = 0.040	H-atom parameters constrained
*wR*(*F*^2^) = 0.107	*w* = 1/[σ^2^(*F*_o_^2^) + (0.0418*P*)^2^ + 0.6807*P*] where *P* = (*F*_o_^2^ + 2*F*_c_^2^)/3
*S* = 1.03	(Δ/σ)_max_ < 0.001
4469 reflections	Δρ_max_ = 0.19 e Å^−^^3^
236 parameters	Δρ_min_ = −0.20 e Å^−^^3^
4 restraints	Extinction correction: *SHELXL97* (Sheldrick, 2008), Fc^*^=kFc[1+0.001xFc^2^λ^3^/sin(2θ)]^-1/4^
Primary atom site location: structure-invariant direct methods	Extinction coefficient: 0.0032 (4)

### Special details

**Table:**

Geometry. All e.s.d.'s (except the e.s.d. in the dihedral angle between two l.s. planes) are estimated using the full covariance matrix. The cell e.s.d.'s are taken into account individually in the estimation of e.s.d.'s in distances, angles and torsion angles; correlations between e.s.d.'s in cell parameters are only used when they are defined by crystal symmetry. An approximate (isotropic) treatment of cell e.s.d.'s is used for estimating e.s.d.'s involving l.s. planes.
Refinement. Refinement of *F*^2^ against ALL reflections. The weighted *R*-factor *wR* and goodness of fit *S* are based on *F*^2^, conventional *R*-factors *R* are based on *F*, with *F* set to zero for negative *F*^2^. The threshold expression of *F*^2^ > σ(*F*^2^) is used only for calculating *R*-factors(gt) *etc*. and is not relevant to the choice of reflections for refinement. *R*-factors based on *F*^2^ are statistically about twice as large as those based on *F*, and *R*- factors based on ALL data will be even larger.

### Fractional atomic coordinates and isotropic or equivalent isotropic displacement parameters (Å^2^)

**Table:**

	*x*	*y*	*z*	*U*_iso_*/*U*_eq_	Occ. (<1)
C1	0.07420 (12)	0.29212 (17)	0.52465 (9)	0.0702 (5)	
H1A	0.0927	0.2750	0.5684	0.105*	
H1B	0.0939	0.2278	0.4960	0.105*	
H1C	0.0132	0.2953	0.5235	0.105*	
C2	0.08842 (9)	0.44770 (14)	0.44314 (7)	0.0447 (3)	
C3	0.03902 (9)	0.38431 (14)	0.39886 (7)	0.0487 (4)	
H3	0.0165	0.3075	0.4099	0.058*	
C4	0.02285 (9)	0.43475 (14)	0.33805 (7)	0.0471 (3)	
H4	−0.0110	0.3917	0.3086	0.056*	
C5	0.05654 (8)	0.54875 (13)	0.32047 (7)	0.0419 (3)	
C6	0.10764 (8)	0.61269 (13)	0.36538 (7)	0.0432 (3)	
H6	0.1313	0.6885	0.3539	0.052*	
C7	0.12280 (9)	0.56397 (14)	0.42603 (7)	0.0448 (3)	
C8	0.20416 (12)	0.73733 (16)	0.46082 (9)	0.0669 (5)	
H8A	0.2364	0.7649	0.4979	0.100*	
H8B	0.1584	0.7942	0.4528	0.100*	
H8C	0.2404	0.7337	0.4232	0.100*	
C10	−0.02731 (9)	0.55919 (13)	0.21288 (7)	0.0432 (3)	
C11	−0.10859 (9)	0.55142 (16)	0.23926 (9)	0.0567 (4)	
H11	−0.1164	0.5629	0.2838	0.068*	
C12	−0.17778 (10)	0.52680 (18)	0.19982 (10)	0.0672 (5)	
H12	−0.2320	0.5229	0.2178	0.081*	
C13	−0.16671 (11)	0.50815 (17)	0.13424 (10)	0.0664 (5)	
H13	−0.2134	0.4912	0.1079	0.080*	
C14	−0.08662 (10)	0.51440 (15)	0.10716 (8)	0.0567 (4)	
H14	−0.0796	0.5011	0.0626	0.068*	
C15	−0.01622 (9)	0.54042 (13)	0.14591 (7)	0.0443 (3)	
C16	0.06748 (10)	0.54873 (14)	0.11162 (7)	0.0496 (4)	
C17	0.13814 (9)	0.47030 (14)	0.13224 (7)	0.0464 (3)	
C9	0.04505 (9)	0.60239 (13)	0.25482 (7)	0.0436 (3)	
O1	0.10795 (7)	0.40790 (11)	0.50401 (5)	0.0615 (3)	
O2	0.17058 (8)	0.61823 (10)	0.47356 (5)	0.0634 (3)	
O3	0.09238 (7)	0.68272 (10)	0.23367 (5)	0.0606 (3)	
O4	0.07382 (8)	0.61470 (13)	0.06358 (6)	0.0795 (4)	
C19	0.22592 (12)	0.31667 (19)	0.17820 (10)	0.0741 (5)	
H19	0.2419	0.2511	0.2050	0.089*	
C20	0.27476 (11)	0.37328 (19)	0.13340 (10)	0.0747 (6)	
H20	0.3309	0.3501	0.1263	0.090*	
S1	0.22947 (5)	0.48657 (9)	0.09150 (7)	0.0691 (3)	0.675 (3)
C18	0.1412 (3)	0.3789 (4)	0.1768 (2)	0.0592 (17)*	0.675 (3)
H18	0.0959	0.3571	0.2036	0.071*	0.675 (3)
S1'	0.13250 (13)	0.3528 (2)	0.18931 (11)	0.0487 (5)	0.325 (3)
C18'	0.2189 (6)	0.4727 (13)	0.1078 (7)	0.160 (9)*	0.325 (3)
H18'	0.2373	0.5316	0.0778	0.192*	0.325 (3)

### Atomic displacement parameters (Å^2^)

**Table:**

	*U*^11^	*U*^22^	*U*^33^	*U*^12^	*U*^13^	*U*^23^
C1	0.0799 (12)	0.0676 (11)	0.0631 (11)	0.0014 (9)	0.0013 (9)	0.0211 (9)
C2	0.0434 (7)	0.0473 (8)	0.0434 (8)	0.0063 (6)	0.0002 (6)	0.0017 (6)
C3	0.0491 (8)	0.0439 (8)	0.0530 (9)	−0.0046 (6)	−0.0002 (7)	0.0035 (7)
C4	0.0457 (8)	0.0474 (8)	0.0481 (8)	−0.0060 (6)	−0.0023 (6)	−0.0029 (7)
C5	0.0387 (7)	0.0450 (8)	0.0420 (7)	0.0002 (6)	0.0029 (6)	−0.0017 (6)
C6	0.0409 (7)	0.0412 (7)	0.0475 (8)	−0.0024 (6)	0.0012 (6)	−0.0024 (6)
C7	0.0410 (7)	0.0457 (8)	0.0477 (8)	0.0011 (6)	−0.0031 (6)	−0.0060 (7)
C8	0.0731 (11)	0.0563 (10)	0.0713 (11)	−0.0111 (9)	−0.0122 (9)	−0.0112 (9)
C10	0.0414 (7)	0.0428 (8)	0.0455 (8)	0.0021 (6)	0.0003 (6)	0.0048 (6)
C11	0.0468 (8)	0.0665 (10)	0.0567 (9)	0.0039 (7)	0.0043 (7)	0.0047 (8)
C12	0.0392 (9)	0.0766 (12)	0.0860 (13)	0.0023 (8)	−0.0010 (8)	0.0088 (10)
C13	0.0522 (10)	0.0669 (11)	0.0801 (13)	0.0023 (8)	−0.0214 (9)	0.0003 (10)
C14	0.0617 (10)	0.0565 (10)	0.0520 (9)	0.0055 (8)	−0.0131 (8)	−0.0006 (8)
C15	0.0479 (8)	0.0403 (7)	0.0448 (8)	0.0039 (6)	−0.0027 (6)	0.0035 (6)
C16	0.0590 (9)	0.0486 (8)	0.0411 (8)	0.0023 (7)	0.0036 (7)	0.0010 (7)
C17	0.0464 (8)	0.0490 (8)	0.0438 (8)	−0.0003 (6)	0.0065 (6)	−0.0059 (7)
C9	0.0436 (7)	0.0431 (7)	0.0441 (8)	0.0003 (6)	0.0049 (6)	−0.0030 (6)
O1	0.0726 (7)	0.0614 (7)	0.0506 (6)	−0.0004 (6)	−0.0104 (5)	0.0102 (5)
O2	0.0760 (8)	0.0576 (7)	0.0565 (7)	−0.0122 (6)	−0.0208 (6)	−0.0018 (5)
O3	0.0676 (7)	0.0615 (7)	0.0527 (6)	−0.0207 (6)	0.0014 (5)	0.0064 (5)
O4	0.0883 (9)	0.0898 (9)	0.0605 (7)	0.0172 (7)	0.0181 (7)	0.0303 (7)
C19	0.0794 (13)	0.0703 (12)	0.0724 (12)	0.0139 (10)	−0.0122 (10)	−0.0096 (10)
C20	0.0453 (9)	0.0834 (13)	0.0954 (15)	0.0117 (9)	0.0010 (10)	−0.0217 (12)
S1	0.0501 (4)	0.0695 (5)	0.0876 (6)	0.0022 (3)	0.0239 (4)	−0.0130 (4)
S1'	0.0502 (9)	0.0473 (9)	0.0485 (9)	0.0120 (7)	0.0026 (7)	0.0027 (8)

### Geometric parameters (Å, º)

**Table:**

C1—O1	1.423 (2)	C11—H11	0.9300
C1—H1A	0.9600	C12—C13	1.370 (3)
C1—H1B	0.9600	C12—H12	0.9300
C1—H1C	0.9600	C13—C14	1.378 (2)
C2—O1	1.3544 (17)	C13—H13	0.9300
C2—C3	1.377 (2)	C14—C15	1.391 (2)
C2—C7	1.411 (2)	C14—H14	0.9300
C3—C4	1.383 (2)	C15—C16	1.495 (2)
C3—H3	0.9300	C16—O4	1.2191 (18)
C4—C5	1.388 (2)	C16—C17	1.460 (2)
C4—H4	0.9300	C17—C18	1.345 (4)
C5—C6	1.4034 (19)	C17—C18'	1.366 (9)
C5—C9	1.4756 (19)	C17—S1	1.6709 (17)
C6—C7	1.370 (2)	C17—S1'	1.728 (3)
C6—H6	0.9300	C9—O3	1.2225 (16)
C7—O2	1.3625 (17)	C19—C20	1.344 (3)
C8—O2	1.415 (2)	C19—C18	1.493 (5)
C8—H8A	0.9600	C19—S1'	1.538 (3)
C8—H8B	0.9600	C19—H19	0.9300
C8—H8C	0.9600	C20—C18'	1.483 (10)
C10—C11	1.391 (2)	C20—S1	1.656 (2)
C10—C15	1.3979 (19)	C20—H20	0.9300
C10—C9	1.501 (2)	C18—H18	0.9300
C11—C12	1.382 (2)	C18'—H18'	0.9300
			
O1—C1—H1A	109.5	C13—C14—H14	119.7
O1—C1—H1B	109.5	C15—C14—H14	119.7
H1A—C1—H1B	109.5	C14—C15—C10	119.35 (14)
O1—C1—H1C	109.5	C14—C15—C16	116.43 (13)
H1A—C1—H1C	109.5	C10—C15—C16	124.22 (13)
H1B—C1—H1C	109.5	O4—C16—C17	120.68 (14)
O1—C2—C3	125.24 (14)	O4—C16—C15	119.11 (14)
O1—C2—C7	115.09 (13)	C17—C16—C15	119.99 (13)
C3—C2—C7	119.67 (13)	C18—C17—C18'	103.3 (6)
C2—C3—C4	120.10 (14)	C18—C17—C16	130.5 (2)
C2—C3—H3	120.0	C18'—C17—C16	126.3 (5)
C4—C3—H3	120.0	C18—C17—S1	112.7 (2)
C3—C4—C5	120.85 (13)	C18'—C17—S1	10.0 (5)
C3—C4—H4	119.6	C16—C17—S1	116.69 (11)
C5—C4—H4	119.6	C18—C17—S1'	5.0 (3)
C4—C5—C6	119.02 (13)	C18'—C17—S1'	108.0 (5)
C4—C5—C9	122.54 (13)	C16—C17—S1'	125.64 (12)
C6—C5—C9	118.33 (12)	S1—C17—S1'	117.35 (12)
C7—C6—C5	120.35 (13)	O3—C9—C5	121.81 (13)
C7—C6—H6	119.8	O3—C9—C10	118.66 (13)
C5—C6—H6	119.8	C5—C9—C10	119.52 (12)
O2—C7—C6	125.38 (13)	C2—O1—C1	117.90 (13)
O2—C7—C2	114.62 (13)	C7—O2—C8	117.72 (12)
C6—C7—C2	120.00 (13)	C20—C19—C18	107.0 (2)
O2—C8—H8A	109.5	C20—C19—S1'	122.1 (2)
O2—C8—H8B	109.5	C18—C19—S1'	15.25 (19)
H8A—C8—H8B	109.5	C20—C19—H19	126.5
O2—C8—H8C	109.5	C18—C19—H19	126.5
H8A—C8—H8C	109.5	S1'—C19—H19	111.3
H8B—C8—H8C	109.5	C19—C20—C18'	103.4 (4)
C11—C10—C15	119.17 (14)	C19—C20—S1	116.37 (14)
C11—C10—C9	119.58 (13)	C18'—C20—S1	13.3 (4)
C15—C10—C9	120.83 (13)	C19—C20—H20	121.8
C12—C11—C10	120.58 (16)	C18'—C20—H20	134.6
C12—C11—H11	119.7	S1—C20—H20	121.8
C10—C11—H11	119.7	C20—S1—C17	91.91 (11)
C13—C12—C11	120.11 (16)	C17—C18—C19	112.0 (3)
C13—C12—H12	119.9	C17—C18—H18	124.0
C11—C12—H12	119.9	C19—C18—H18	124.0
C12—C13—C14	120.26 (16)	C19—S1'—C17	92.13 (16)
C12—C13—H13	119.9	C17—C18'—C20	114.1 (8)
C14—C13—H13	119.9	C17—C18'—H18'	123.0
C13—C14—C15	120.52 (16)	C20—C18'—H18'	123.0
			
O1—C2—C3—C4	179.55 (13)	C4—C5—C9—C10	21.6 (2)
C7—C2—C3—C4	−0.3 (2)	C6—C5—C9—C10	−162.15 (12)
C2—C3—C4—C5	0.6 (2)	C11—C10—C9—O3	−131.30 (15)
C3—C4—C5—C6	0.1 (2)	C15—C10—C9—O3	41.2 (2)
C3—C4—C5—C9	176.30 (13)	C11—C10—C9—C5	47.76 (19)
C4—C5—C6—C7	−1.1 (2)	C15—C10—C9—C5	−139.72 (14)
C9—C5—C6—C7	−177.44 (12)	C3—C2—O1—C1	−0.8 (2)
C5—C6—C7—O2	−179.37 (13)	C7—C2—O1—C1	179.09 (14)
C5—C6—C7—C2	1.3 (2)	C6—C7—O2—C8	3.2 (2)
O1—C2—C7—O2	0.10 (18)	C2—C7—O2—C8	−177.42 (14)
C3—C2—C7—O2	179.99 (13)	C18—C19—C20—C18'	2.3 (7)
O1—C2—C7—C6	179.46 (12)	S1'—C19—C20—C18'	4.5 (7)
C3—C2—C7—C6	−0.6 (2)	C18—C19—C20—S1	−1.0 (3)
C15—C10—C11—C12	−0.7 (2)	S1'—C19—C20—S1	1.2 (3)
C9—C10—C11—C12	171.96 (15)	C19—C20—S1—C17	1.00 (17)
C10—C11—C12—C13	0.9 (3)	C18'—C20—S1—C17	−13 (3)
C11—C12—C13—C14	−0.3 (3)	C18—C17—S1—C20	−0.7 (3)
C12—C13—C14—C15	−0.4 (3)	C18'—C17—S1—C20	19 (4)
C13—C14—C15—C10	0.5 (2)	C16—C17—S1—C20	−176.59 (13)
C13—C14—C15—C16	−178.31 (15)	S1'—C17—S1—C20	−2.63 (14)
C11—C10—C15—C14	0.0 (2)	C18'—C17—C18—C19	−3.2 (8)
C9—C10—C15—C14	−172.56 (13)	C16—C17—C18—C19	175.46 (19)
C11—C10—C15—C16	178.76 (14)	S1—C17—C18—C19	0.3 (4)
C9—C10—C15—C16	6.2 (2)	S1'—C17—C18—C19	160 (3)
C14—C15—C16—O4	50.8 (2)	C20—C19—C18—C17	0.4 (4)
C10—C15—C16—O4	−127.95 (17)	S1'—C19—C18—C17	−172.7 (12)
C14—C15—C16—C17	−123.81 (15)	C20—C19—S1'—C17	−2.5 (2)
C10—C15—C16—C17	57.4 (2)	C18—C19—S1'—C17	5.3 (9)
O4—C16—C17—C18	−169.2 (3)	C18—C17—S1'—C19	−18 (3)
C15—C16—C17—C18	5.4 (4)	C18'—C17—S1'—C19	−0.7 (8)
O4—C16—C17—C18'	9.2 (9)	C16—C17—S1'—C19	176.50 (15)
C15—C16—C17—C18'	−176.3 (9)	S1—C17—S1'—C19	3.15 (16)
O4—C16—C17—S1	5.9 (2)	C18—C17—C18'—C20	4.8 (13)
C15—C16—C17—S1	−179.57 (11)	C16—C17—C18'—C20	−173.9 (6)
O4—C16—C17—S1'	−167.53 (16)	S1—C17—C18'—C20	−157 (5)
C15—C16—C17—S1'	7.0 (2)	S1'—C17—C18'—C20	3.3 (13)
C4—C5—C9—O3	−159.37 (14)	C19—C20—C18'—C17	−4.7 (13)
C6—C5—C9—O3	16.9 (2)	S1—C20—C18'—C17	162 (4)

### Hydrogen-bond geometry (Å, º)

Cg2 is the centroid of the C17/C19/C20/S1'/C18' ring.

**Table:**

*D*—H···*A*	*D*—H	H···*A*	*D*···*A*	*D*—H···*A*
C3—H3···O4^i^	0.93	2.58	3.496 (2)	169
C1—H1*A*···*Cg*2^ii^	0.96	2.99	3.799 (3)	143

Symmetry codes: (i) −*x*, *y*−1/2, −*z*+1/2; (ii) −*x*−1/2, *y*−1/2, *z*.
